# UNDERSTANDING NON-MEDICAL COSTS FOR HEALTH CARE: EVIDENCE FROM INPATIENT CARE FOR OLDER PEOPLE IN CHINA

**DOI:** 10.1093/geroni/igz038.2686

**Published:** 2019-11-08

**Authors:** Wei Yang

**Affiliations:** King’s College London, London, United Kingdom

## Abstract

Non-medical costs can constitute a substantial part of total health care costs, especially for older people. Costs associated with carers, travel, food and accommodation for family members accompanying and caring for older people during their medical visits can be hefty. This study seeks to examine the effects of non-medical costs on catastrophic health payments and health payment-induced poverty among older people in rural and urban China. Using data from the China Health and Retirement Longitudinal Survey 2015, this study finds that inpatient costs account for a significant proportion of household expenditure, and non-medical costs can account for approximately 18% of total costs. That share is highest for those who belong to the lowest wealth groups. Non-medical costs increase the chances of older people incurring catastrophic health payments and suffering from health payment-induced poverty. Such effects are more concentrated among the poor than the rich. The results also show that the rural population are more likely to incur catastrophic health payments and suffer from health payment induced poverty compared to the urban population. This paper urges policy makers to consider reimbursing the non-medical costs of patient care, improving health care systems in general and for the rural populations specifically.

